# Early discharge criteria in patients with acute bacterial skin and skin structure infections in a large tertiary-care teaching hospital in Florence, Italy

**DOI:** 10.1007/s10096-019-03609-9

**Published:** 2019-06-20

**Authors:** Filippo Lagi, Letizia Ottino, Elisabetta Mantengoli, Alberto Distefano, Giampaolo Corti, Alberto Farese, Bassam Dannaoui, Alessandra Ipponi, Tiziana Falai, Gian Maria Rossolini, Alessandro Bartoloni, Filippo Bartalesi

**Affiliations:** 1 Department of Experimental and Clinical Medicine, University of Florence, Florence, Italy; 2 Infectious and Tropical Diseases Unit, Careggi University Hospital, Florence, Italy; 3 Health Direction, Careggi University Hospital, Florence, Italy; 4 Hospital Pharmacy and Pharmaceutical Policies, Careggi University Hospital, Florence, Italy; 5 Clinical Microbiology and Virology Unit, Careggi University Hospital, Florence, Italy

**Keywords:** ABSSSI, Early discharge, Long-acting, Italy

## Abstract

The study is aimed at retrospectively estimating the percentage of inpatients with severe acute bacterial skin and skin structure infections (ABSSSI) who met the early discharged (ED) criteria adapted from Nathwani et al. (Int J Antimicrob Agents. 2016 Aug;48(2):127-36) and to calculate the number of hospitalization days that could be potentially saved. A retrospective study was conducted in a tertiary care hospital in Florence, Italy. We included all patients admitted for cellulitis and post-surgical infections from 2014 to 2017. Demographic and clinical data were obtained from electronic medical records. We a priori defined the following as a risk factor for non-adherence (RFNA): active or on methadone intravenous drug users, homeless, migrants without health care assistance, and patients who need a caregiver to take prescribed medications. One hundred sixty-two subjects were enrolled. Of them, 94 (58.0%) were male, and 113 (69.7%) had cellulitis/erysipelas. A microbiological isolate was obtained in 51 patients (31.4%); *Staphylococcus aureus* was the most frequent (47%). Eighty-four (51.8%) were ED suitable, with 258 (49.0%) patient days potentially saved. Among the 78 not ED suitable patients, the most common reason for prolonged length of stay (LOS) was having at least one RFNA (34.6%). Fourteen (18.0%) had one RFNA. Half of the patients admitted in our hospital met the ED criteria with a sparing close to 50% in terms of hospitalization days. Unstable social and personal factors were the most frequent causes for prolonged LOS. In this selected subset of patients, more recent and easier to administer treatments, including long-acting agents, could be proposed.

## Background

In 2013, the United States (US) Food and Drug Administration (FDA) proposed new guidelines and recommendation on developing drugs for the treatment of skin and soft tissues infections, using a new definition of acute bacterial skin and skin structure infection (ABSSSI) [1]. According to FDA, ABSSSI is defined as a bacterial infection of the skin with a lesion size area of at least 75 cm^2^ and the infection types included are cellulitis, erysipelas, major skin abscesses, and wound infections [1, 2].

ABSSSI is a frequent cause of morbidity and represents a frequent cause of admission to the emergency department [3]. The most common pathogens involved in ABSSSI are typical skin colonizers such as Gram-positive cocci and, in particular, beta-hemolytic streptococci and *Staphylococcus aureus* [4]. Globally, the high level of resistance to many antibiotics in *S*. *aureus* makes the appropriate selection of the antibiotic for physicians highly challenging and a severe threat for patients [5]. Antibiotic-resistant bacteria such as methicillin-resistant *S*. *aureus* (MRSA) causing ABSSSI are more challenging to treat due to the lower availability of effective antibiotics and higher mortality rate [5]. The management of ABSSSI often requires intravenous antibiotic therapy and infection-source control through surgical debridement or abscess drainage [6].

The need for intravenous antibiotic therapy, along with an increase of drug-resistant bacteria such as MRSA, both impact on the duration ofhospitalization causing high costs and increasing patient’s morbidity and mortality [7, 8]. Length of hospitalization could be shortened when patients are suitable for early discharge according to standardized clinical criteria for switching to an oral antibiotic [9]. However, in some cases, it could not be feasible because of unstable social and personal factors that would interfere with successful outpatient care [3]. New developed long-acting antibiotics with MRSA activity are recently approved for ABSSSI treatment and could be suitable in this subset of patients.

We report the result of a study conducted in an Italian tertiary care teaching hospital focused on ABSSSI with the purpose to estimate the percentage of inpatients with ABSSSI who could be discharged earlier, based on adapted criteria from Nathwani et al. and calculate the number of inpatient day that could have been saved [9]. We also investigated factors associated with prolonged length of stay.

## Methods

### Study setting and patients

We conducted a retrospective cross-sectional study in Azienda Ospedaliero-Universitaria Careggi (Careggi Hospital), Florence, Italy. Careggi Hospital is a tertiary care teaching hospital with 1297 beds and more than 60,000 hospitalizations per year. From January 2015, a multidisciplinary antimicrobial stewardship team operates in Careggi Hospital ensuring the proper use of antimicrobial agents and the adherence to protocols for infection management. However, no defined protocol for ABSSSI was yet in place at the time of the study. The local ethical committee approved the study with the reference number: 11689_oss.

We enrolled all adult patients (≧ 18 years) hospitalized for cellulitis and surgical site infections selected by diagnosis-related group (DRG) from January 1, 2014 to December 31, 2017. The DRG codes included in the analysis were: 277 (cellulitis > 17 years with complications), 278 (cellulitis > 17 years without complications), and 418 (post-surgical and traumatic infections). Demographic, clinical, and laboratory characteristics were extrapolated from electronic medical records on the day of the first evaluation in the emergency department, and on the third day (+ 1 day if no laboratory or clinical data were available in the medical records) of hospitalization. By ABSSSI definition, all patients with one or more of the following conditions were excluded from the analysis: impetigo, burns, minor abscess, bites, necrotizing lesions, diabetic foot, chronic ulcers, septic arthritis, osteomyelitis, pregnancy, neutropenia defined as white blood cells < 1000 cells/mm^3^, and clean contaminated/contaminated wounds surgery. If a patient had more than one hospitalization in the last 12 months, we included only the first episode.

### Early discharge criteria and interpretation

A patient was eligible for early discharge if all the criteria listed in Table 1 were present on day 3 (+ 1) of hospitalization. The proportion of hospitalized patients meeting the adapted early discharge criteria from Nathwani et al. was assessed using a dichotomous variable that expressed adherence to the criteria on day 3 (+ 1 day). The population was then split into two groups: patients who met early discharge criteria (ED group) and who did not (NED group). The days of hospitalization in excess in ED group criteria were calculated.

**Table 1:** Patient eligibility criteria for early discharge in ABSSSI patients (adapted from Nathwani et al.) [9]

I.v. antibiotics for > 24 h	
Afebrile (temperature < 38 °C) for > 24 h	
Clinical lesion improvement of the lesion or stable infection	
WBC count of 4 × 10^9^/L to 11 × 10^9^/L	
No unexplained tachycardia	
Systolic blood pressure ≥ 100 mmHg	
Patient tolerates oral fluids/diet and can take oral medications with no gastrointestinal absorption problems	
No other reason to stay in hospital except for infection management (e.g., surgery scheduled in 48 h; the presence of drainage)	
Stable co-morbid illness	
No risk factors for non-adherence for the withdrawal and the correct self-administration of home oral therapy: • Active or former intravenous drug users receiving methadone • Homeless • Migrants without health care assistance • Patients who need a caregiver because not able to take prescribed medications on their own • Patients with vomit/diarrhea or any other medical condition that makes them unable to swallow	

*i*.*v*., intravenous; *WBC*, white blood cell; *ABSSSI*, acute bacterial skin and skin structure infections

When lesion size measurement on day 3 was not reported in medical records, we considered the expression “lesion’s clinical improvement” equivalent to the reduction of at least 20% in lesion size. A stable social and mental condition was interpreted as the possibility of autonomously providing for the withdrawal and correct self-administration of home therapy. We a priori considered the following situations a risk factor for non-adherence: active intravenous drug users (IVDU) or on methadone treatment, homeless, migrants without health care assistance, and patients who need a caregiver due to inability to take prescribed medications on their own (e.g., elderly people with inability for self-care or home transfer, and subjects with psychiatric illness with severe degree of disability). Finally, we considered patients with vomit/diarrhea or any other medical condition that made them unable to swallow (e.g., esophageal or neurological disorders) not eligible for early discharge (Table 1).

### Statistical methods

Data were analyzed using the Stata/MP version 14.0 program (StataCorp, College Station, TX). The continuous variables were expressed with median and interquartile range, categorical variables as proportions. The differences between the groups were assayed by Student *t* or Wilcoxon ranks test where appropriate.

#### Availability of data and material

The datasets used and/or analyzed during the current study are available from the corresponding author on reasonable request.

## Results

We evaluated 229 patients. According to the FDA’s ABSSSI definition, 67 patients were excluded (21 immunosuppressed, 13 diabetic foot, 9 missing data, 5 osteomyelitis, 7 prosthetic joint infections, 6 contaminated surgery wound infections, 4 animal bites, 2 pregnancy). The size of the lesion was reported only in nearly 20% of the medical records. Finally, 162 subjects were included. Demographic and clinical characteristics of the study population are summarized in Table 2. A microbiological isolate was obtained in 51 subjects (31.4%), and *S*. *aureus* was the most frequent isolate (47.0%): methicillin-susceptible *S*. *aureus* (MSSA) in 17 cases (70.8%) and MRSA in 7 (29.2%). Other isolates were: *Streptococcus pyogenes* (*N* = 4; 7.8%), *Streptococcus* spp. (*N* = 6; 8.5%)*,* coagulase-negative staphylococci (*N* = 4; 7.8%), *Enterococcus* spp. (*N* = 4; 7.8%), *Escherichia coli* (*N* = 2; 3.9%), *Pseudomonas aeruginosa* (*N* = 3; 5.9%), and polymicrobial (*N* = 4; 7.8%).

**Table 2:** Demographic and clinical characteristics of the ABSSSI population (total *N* = 162)

	*N* (%)
Male	94 (58.0)
Female	68 (42.0)
Age, median (IQR)	61 (43–78)
Type of lesion	
• Cellulitis/erysipelas	113 (69.7)
• Wound infection	28 (17.3)
• Major cutaneous abscess	21 (13.0)
Location of ABSSSI	
• Lower limb	85 (52.5)
• Upper limb (no hand)	16 (9.0) (9.8)
• Hand	6 (3.7)
• Face	28 (17.3)
• Groin/sacral area	10 (6.2)
• Abdomen	7 (4.3)
• Chest	10 (6.2)
Previous episode of ABSSSI	
• No	148 (91.0) (91.4)
• Yes	14 (8.6)
Number of hospitalization in the previous 12 months	
• 0	97 (59.9)
• ≥ 1	65 (40.1)
Charlson comorbidity index	
• 0–1	114 (70.4)
• ≥ 2	48 (29.6)
Previous antibiotic therapy	
• No	100 (61.7)
• Yes	62 (38.3)
Number of concomitant medications	
• 0–1	59 (36.4)
• 2–3	31 (19.0) (19.2)
• > 4	72 (44.4)
Median days of hospitalization (IQR)	7 (4–10)
Antibiotic therapy at discharge	
• No	25 (15.4)
• Yes	137 (84.6)
Empiric anti-MRSA therapy	
• No	102 (63.0)
• Yes	60 (37.0)

*ABSSSI* (Acute bacterial skin and skin structure infections); *MRSA* (methicillin-resistant S.aureus)

Eighty-four (51.8%) patients were suitable for early discharge (ED group), and 16 of them (19.0%) had a length of stay (LOS) ≤ 3 days. In the ED group, we recorded 527 days of hospitalization with 258 days (49.0%) that could have been potentially saved (median 3 days per patient). Among the 78 patients not suitable (48.2%) for the early discharge (NED group), the most frequent reason for prolonged LOS was to have at least one risk factor for non-adherence (27 patients, 34.6%, Table 3). In the NED group, 14 (18.0%) patients had the sole presence of a risk factor for non-adherence. No difference in age or Charlson comorbidity index was observed between ED and NED groups. Median LOS was 6 (IQR 4–8) days and 9 (IQR 7–12) days in the ED and NED group, respectively (*p* < 0.001).

**Table 3:** Frequency of reasons for not meeting discharge criteria in patients with ABSSSI

Presence of at least one risk factor for non-adherence	27 (34.6)
WBC count of 4 × 10^9^/L to 10 × 10^9^/L	24 (30.7)
No lesion improvement or stable infection	19 (24.4)
Febrile (temperature > 38 °C) for > 24 h	17 (21.8)
Other reasons to stay in hospital (e.g., surgery scheduled in 48 h; the presence of drainage)	13 (16.7)
Unstable co-morbid illness	11 (14.1)
A patient does not tolerate oral fluids/diet or/and GI absorption problems	5 (6.4)
I.v. antibiotics for < 24 h	3 (3.8)
Unexplained tachycardia	1 (1.3)

*i*.*v*., intravenous; *WBC*, white blood cell; *ABSSSI*, acute bacterial skin and skin structure infections

## Discussion

Applying adapted Nathwani et al. criteria to 162 ABSSSI patients observed in our hospital, 285 patient days could have been saved with a median per patient of 3 days (IQR 1–5). To the best of our knowledge, only the study conducted by Eckmann et al. assessed early switch and early discharge criteria in skin and soft tissue infections in Europe [10]. Although, the Eckmann et al. and our study are scarcely comparable because of a different population enrolled in terms of site of infection (complicated skin and soft tissue infections vs. ABSSSI) and etiology (MRSA vs. all other etiology), our analysis showed a higher percentage of eligible patients for early discharge (34.2% vs. 51.8%).

Unfortunately, we cannot determine the level of awareness regarding early discharge criteria among physicians and the retrospective design of our study did not permit to clearly identify the reasons for a missed early discharge in the ED group.

Moreover, the premise of early discharge is not tied only to the clinical improvement and to the patient’s ability to take the oral prescribed medications but also depends on the ability to provide supply, preparation, and administration of home therapy autonomously. This aspect, often underestimated in switch studies, appeared to be fundamental in the population analyzed, where the most frequent reason for a prolonged LOS in the NED group was the presence of at least one risk factor for non-adherence. IVDU and a psychiatric pathology resulting in a severe degree of disability were the two most common risk factors for non-adherence with 11.5% and 8.9%, respectively [11]. Patients with IV drug addiction or severe psychiatric conditions (e.g., delirium, dementia, and depressed mood) are known to have an extended hospitalization [12, 13]. Furthermore, an early discharge in IVDU would offer the advantage of the quick removal of the IV access sometime used for illicit drug injection even in a hospital setting. Interestingly, in the NED group, 14 (18.0%) patients had no clinical reason to stay in the hospital and were not discharged because of the sole presence of a risk factor for non-adherence.

In this perspective, new long-acting therapeutic possibilities (dalbavancin, oritavancin) recently approved for ABSSSI by the European Medicines Agency (EMA) and the FDA could notably reduce the risk of non-compliance with the antibiotic therapy because of their simple dosage and excellent tolerability [7]. Due to their simple administration and greater tissue penetration, they could alleviate the need for a prolonged hospitalization, especially in those patients that are unreliable with regard to following treatment instructions and meeting follow-up needs or have any medical condition that makes them unable to swallow. Finally, if administrated in the very early phase, it may reduce the need for the placement of indwelling catheters especially in population like IVDU potentially at risk for misuse of the device [7]. Although the initial expense for these new types of long-acting drugs proves to be greater than other solutions, cost-effectiveness studies have shown that the costs are amortized by reducing the need of hospitalization and LOS [14, 15]. While accurate financial accounting was not possible as part of this study, we envisage substantial savings with this approach. This result has been discussed and endorsed by physicians from infectious diseases, emergency department, internal medicine, surgery, health directorate as well as microbiologist and pharmacists. An internal procedure for ABSSSI management was finally implemented.

This study has several limitations mainly due to its retrospective nature, which could have carried to misclassification of patient exposure and outcome. In fact, a priori criteria could overestimate the true prevalence of risk factors for non-adherence. On the other hand, the proportion of some risk factors could have been underestimated because of a missing report in the medical records (e.g., family and social situation).

## Conclusions

To the best of our knowledge, our research fills a gap in the literature by providing an Italian real-life assessment of ABSSSI, irrespective from the microbiological isolation and taking into the account social and mental situation. Our study showed that about half of the patients admitted met the criteria for an early discharge with a sparing of nearly 50% in terms of hospitalization days. In our opinion, raising awareness among the clinicians on adherence to early discharge criteria may lead to a reduction in the timing of hospitalization. The decline in hospitalization time would improve the patient’s quality of life and satisfaction, decrease the risk of contracting an intra-hospital infection, and get a reduction in hospital costs, thus going to strengthen and rationalize health spending efficiently. The recent availability of new antibiotics with a better safety profile and easy dosage would ensure appropriate treatment at home after an early discharge also in populations with social problems and not inserted in contexts of support.

## Abbreviations

ABSSSI: acute bacterial skin and skin-structure infection
DRG: diagnosis-related group
ED: early discharge
ED group: patients who met early discharge criteria
FDA: Food and Drug Administration
IVDU: intravenous drug users
MRSA: methicillin-resistant *S*. *aureus*
MSSA: methicillin-susceptible *S*. *aureus*
NED group: patients who did not meet early discharge criteria
